# Insights into Mechanisms and Proteomic Characterisation of *Pseudomonas aeruginosa* Adaptation to a Novel Antimicrobial Substance

**DOI:** 10.1371/journal.pone.0066862

**Published:** 2013-07-15

**Authors:** Peter Cierniak, Martin Jübner, Stefan Müller, Katja Bender

**Affiliations:** 1 Institute of Legal Medicine, Medical Faculty, University of Cologne, Cologne, Germany; 2 Center for Molecular Medicine Cologne, University of Cologne, Cologne, Germany; NIAID, United States of America

## Abstract

Antibiotic resistance has been reported since the introduction of synthetic antibiotics. Bacteria, such as one of the most common nosocomial pathogens *P. aeruginosa*, adapt quickly to changing environmental conditions, due to their short generation time. Thus microevolutional changes can be monitored *in situ*. In this study, the microevolutional process of *Pseudomonas aeruginosa* PAO1 resistance against a recently developed novel antibacterial zinc Schiff-base (ZSB) was investigated at the proteome level. After extended exposure to ZSB the passaged strain differed in tolerance against ZSB, with the adapted *P. aeruginosa* PAO1 exhibiting 1.6 times higher minimal inhibitory concentration. Using Two-dimensional Difference Gel Electrophoresis, the changes in the proteome of ZSB adapted *P. aeruginosa* PAO1 were examined by comparison with the non-adapted *P. aeruginosa* PAO1. The proteome of the adapted *P. aeruginosa* PAO1 strain differed significantly from the non-adapted in the abundance of two proteins when both strains were grown under stressing conditions. One protein could be identified as the outer membrane protein D that plays a role in uptake of basic amino acids as well as in carbapeneme resistance. The second protein has been identified as alkyl peroxide reductase subunit F. Our data indicated a slight increase in abundance of alkyl peroxide reductase F (AhpF) in the case of ZSB passaged *P. aeruginosa* PAO1. Higher abundance of Ahp has been discussed in the literature as a promoter of accelerated detoxification of benzene derivatives. The observed up-regulated AhpF thus appears to be connected to an increased tolerance against ZSB. Changes in the abundance of proteins connected to oxidative stress were also found after short-time exposure of *P. aeruginosa* PAO1 to the ZSB. Furthermore, adapted *P. aeruginosa* PAO1 showed increased tolerance against hydrogen peroxide and, in addition, showed accelerated degradation of ZSB, as determined by HPLC measurements.

## Introduction

Microevolution is defined as an adaptive modification in a population over a given time that occurs as a continuous natural process with gradual change [1]. The gradual change is caused, as observed by Charles Darwin and Alfred Russel Wallace, by different principles: 1) All individual variations among organisms are propagated vertically from parental to the next generation. 2) All organisms generate more offspring than necessary to replace themselves. 3) Environmental changes cause a distinct pressure by limiting the survivability of a population, which controls the population size. 4) Individuals who are still able to survive and generate offspring within those changed conditions predominantly take benefit of their individual variations, and are better adapted to the changed conditions than the non-survivors [1]–[3]. In contrast to many multicellular organisms many bacteria have very short generation times. It is therefore possible to follow the microevolution in a bacterial population described by the named principles *in situ*, thus making predictions on population changes possible [4].

An alarming example of adaptation processes is the rapid onset in resistance of bacteria against antibiotics or biocides [5]. Resistances were reported soon after the introduction of synthetic antibiotics in the early twentieth century [6], and these resistances are on the increase [7]. Different modes of acquired and intrinsic resistance are known. One mode is the alteration of the antibiotic drug target; such as changes in the ribosome structure [8]. Another mode of resistance is decreased membrane permeability, thereby preventing the uptake of the drug [9]. Over-expression of efflux systems that lead to removal of the antibacterial drug is another general mode of resistance [10]. A prominent mechanism of resistance to antibiotics is the enzymatic destruction of the drug by enzymes such as beta-lactamases [11].

The emergence of multi-drug-resistant bacteria and the development of new antibacterial substances have developed into a race that is currently driven by the continuous introduction of new antimicrobial substances that exhibit completely new mechanisms of drug action. To minimize the spread of germs, elevated research activities in developing antimicrobial surfaces have led to diverse antibacterial materials, such as metal-containing coatings or antibacterial polymers [12]. Due to the increasing interest in antimicrobial materials as surface impregnations, attention should also be paid to the evolution of resistances against these materials. Besides silver or copper, other metals, such as zinc, show antimicrobial properties (reviewed in [13]). Thus zinc-containing materials are currently under investigation in the production, and for use as, antimicrobial surfaces – both for the protection of medical devices as well as household items. The aim of our study was to elucidate the possibility of resistance development against zinc-containing compounds as an antimicrobial material, and to gain knowledge of the underlying mechanism.

Recently, various Schiff-base derivatives have aroused considerable interest [14]–[17] due to their antibacterial activities [15], offering a source for antibacterial substances. The Schiff-base bis(N-allylsalicylideneiminato)-zinc (ZSB), developed, synthesized and characterized by Poulter and colleagues, demonstrated antibacterial properties, when deposited on surfaces by plasma-enhanced chemical vapour deposition (PECVP) [15]. In our study the same ZSB was used as a prototype of a new antibacterial material, to investigate microevolution.

*Pseudomonae* are a ubiquitous genus of gram-negative bacteria that occur in terrestrial as well as in aquatic environments. They are found associated together with plants as well as animals, emphasising the versatility of this genus, both genetically and metabolically (reviewed in [18]). *P. aeruginosa* is a nosocomial pathogen that accounts for around a third of all intensive care unit infections in Europe [19]. It is an opportunistic pathogen that causes infections in immuno-compromised persons [20], and is associated with cystic fibrosis with a prevalence of more than 80% [21], as well as with burn wound infections [22]. *Pseudomonas aeruginosa* PAO1 was used as the prototype bacterial strain for microevolution experiments.

In this study we present data on the adaptation of *P. aeruginosa* PAO1 to ZSB for nearly 300 generations. The adapted strain was compared to its non-adapted, ancestral strain by Two-dimensional Difference Gel Electrophoresis (2-D DIGE) and mass spectroscopy. The results of the proteomic work were verified by comparison of the physiological behaviour of the adapted and the non-adapted *P. aeruginosa*. Based on this data, we demonstrate the formation of tolerances by means of natural selection in *P. aeruginosa* PAO1. Tolerance comes along with accelerated degradation of the antibacterial compound. Except for directed genetic modifications [23] [24] the presented study is, to our knowledge, the only study that has monitored evolutional changes in stressed bacteria at the proteome level against a newly synthesised antimicrobial substance.

## Materials and Methods

### Bacterial strain and culture conditions

*Pseudomonas aeruginosa* PAO1 (kindly provided by A. T. A. Jenkins, University of Bath, UK) was routinely grown in Lysogeny Broth [25], [26] (Luria/Miller, LB, Carl Roth, Karlsruhe, Germany) at 37°C in an orbital shaking incubator (GFL, Burgwedel, Germany) at 170 rpm, unless otherwise stated. Stock cultures were stored in 20% (v/v) glycerol at −80°C.

### Antibacterial agents

The zinc Schiff-base complex bis(N-allylsalicylideneiminato)-zinc (ZSB), (MW 771 g mol^−1^) was synthesized and characterized by Poulter et al. [15]. The compound consists of two zinc atoms chelated by four allylsalicylideneimin Schiff-base (SB) ligands. ZSB and the zinc free ligand SB were provided by Poulter and colleagues, University of Bath, UK. ZSB and SB were dissolved in >99.99% dimethyl sulfoxide (DMSO) (Sigma-Aldrich; Sigma-Aldrich Chemie GmbH, Taufkirchen Germany) respectively and diluted in LB medium to a final DMSO concentration of 2% (v/v).

### Adaptation of *P. aeruginosa* PAO1 in zinc Schiff-base

Microevolution was achieved according to a modified protocol of Lenski and colleagues [4]. The experimental setup for the microevolution with ZSB included 3 to 6 bacterial cultures per passage. The cultures were grown under continuous agitation at 170 rpm for 20 to 48 h in glass tubes containing 6 mL LB medium with a distinct concentration of ZSB. Starting concentration for the first passages was 0.5 mM of ZSB, which was increased stepwise by 0.2 mM, and in the final step by 0.4 mM, up to 1.3 mM in total. To monitor the growth of the bacteria, the optical density was measured after 20 to 48 h of cultivation at wavelength λ 600 nm (OD_600_) (Geneys UV10, Thermo Fisher Scientific Germany Ltd. & Co. KG, Braunschweig, Germany). After each incubation period, the culture with the highest OD_600_ was used for the next passage. Additionally, in order to make comparison with the ancestral cultures possible, at the start of the experiment and at every 5–10^th^ passage, glycerol cultures were prepared and stored at −80°C. The ZSB Stock solutions were exchanged every 5^th^ to 10^th^ passage. The initial viable cell number before incubation of each passage was 10^7^ CFU mL^−1^, and after incubation 10^9^ CFU mL^−1^. In total, the microevolution was performed over almost 300 generations (45 passages) until significant adaptation was reached.

The purity of the cultures was tested after every 8^th^ –10^th^ passage, in order to detect contamination. LB agar and Cetrimide agar (Sigma-Aldrich Chemie GmbH, Taufkirchen, Germany) plates were inoculated with the respective passaged liquid culture and incubated for 24 h at 37°C. The culture was only utilised for further passages if a single colony type could be identified on LB agar, and no change between the passaged and the non-passaged *P. aeruginosa* was found on Cetrimide agar with respect to colony appearance, pigmentation and fluorescence at 254 nm.

To verify whether the ZSB adapted strain was able to grow in the presence of significantly higher concentrations of ZSB, both the non-adapted cultures and the ZSB adapted cultures were grown in LB medium at increasing concentrations of ZSB for 23 to 24 h. Prior to the experiments, in order to reduce the influence of metabolic modifications, the non-adapted culture was incubated for one passage at 0.2 mM of the ZSB. The adapted and the preconditioned non-adapted cultures were diluted with LB medium to OD_600_ =  1. Then 6 mL of LB medium containing 2% (v/v) DMSO and a defined concentration of ZSB was inoculated with 60 µL of the prepared bacteria dilutions. After incubation the OD_600_ was measured. All experiments were prepared in duplicate on two separate days. LB medium containing 2% (v/v) of DMSO was used as a control.

In order to verify whether the increased tolerance to ZSB was due to amplified metabolic activity or to adaptation, the *P. aeruginosa* PAO1 P_45_ strain was grown for two passages in ZSB-free LB medium (P_45_L). The OD_600_ of the non-adapted P_0_, the initial adapted (P_45_) and the P_45_L strain were compared after incubation in LB medium containing 1.3 mM ZSB at 24 h intervals for up to 96 h.

### Proteomic analysis of adapted and non-adapted *P. aeruginosa* PAO1

Preparation of Bacterial cultures: To allow the comparison of the proteomes from the adapted P_45_ and the non-adapted *P. aeruginosa* PAO1, conditions were defined in which both bacteria were able to grow at similar velocities to avoid artefacts arising from different metabolic activities of the cultures. Cultures were grown in baffled Erlenmeyer flasks in 20 mL ZSB-free LB medium for three to four hours, until the cultures reached an OD_600_ of 0.65, after which ZSB was added to a final concentration of 0.5 mM to both adapted and non-adapted *P. aeruginosa* PAO1. Once both experimental groups had reached an OD_600_ of 0.9, the samples were harvested by centrifugation at 5000 *g* for 10 min at 23°C (Biofuge Primo R, Thermo Fisher Scientific Germany Ltd. & Co. KG, Braunschweig, Germany). Pellets were washed three times with PBS pH 7.6 (Carl Roth, Karlsruhe, Germany) and stored at −80°C until use. Each experimental group consisted of four biological replicates.

Comparative proteome analysis of adapted and non-adapted strains: Proteomic analysis was performed, with some minor adjustments (listed below), according to the protocols from A. Görg and colleagues [27] [28]. The protein pellets were thawed on ice and resuspended in 1 mL lysis buffer (7 M urea, 2 M thiourea, 30 mM TRIS (pH 7.5), 4% v/v CHAPS) containing a final concentration of 1 x nuclease mix and 1 x protease inhibitor. The samples were homogenized by ultrasonication (Bandelin electronic, Berlin, Germany) on ice at 40% cycle rate and 13% amplitude for 1 min and subsequently incubated for 15 min at room temperature. The samples were centrifuged at 8000 *g* for 20 min at 4°C to remove insoluble material after incubation. The supernatant was then transferred into 1.5 mL centrifuge tubes and centrifuged again at 25000 *g* for 45 min at 4°C. Subsequently, proteins were isolated from the crude extracts by the 2-D Clean-Up Kit (GE Healthcare, Uppsala, Sweden) according to manufacturer's instructions and dissolved in lysis buffer (7 M urea, 2 M thiourea, 4% (v/v) CHAPS, 30 mM tris (pH 7.5)). The pH of the dissolved samples was adjusted to 8.5 on ice with 0.1 M NaOH (analytical grade, Carl Roth, Karlsruhe, Germany) and the protein concentration was photometrically determined using the 2-D Quant kit (GE Healthcare, Uppsala, Sweden). The proteins of the samples were then labelled by applying the CyDye™ DIGE Fluor minimal labelling kit (GE Healthcare, Uppsala, Sweden) according to manufacturer's instructions. After labelling, the samples were mixed 1∶1 with sample buffer (7 M urea, 2 M thiourea, 4% (v/v) CHAPS, 2% (v/v) ampholytes pH 4–7) and pooled. Each pooled sample consisted of an assay of the non-adapted group, an assay of the adapted group and a mixture of all samples (internal standard). The dye order was swapped intentionally from one sample to the next. The samples were then applied cathodically onto an immobilized pH gradient (IPG) 18 cm stripe, pH 4–7 (GE Healthcare, Uppsala, Sweden). Samples were separated in the second dimension (Ettan DALTsix, GE Healthcare, Uppsala, Sweden) for 5 h. A fluorescent scanner equipped with lasers emitting three different wavelength, with associated band pass filters, was used to scan the gels (Typhoon Trio, GE Healthcare, Uppsala, Sweden). Experimental groups were analysed by DeCyder™ V6 (GE Healthcare, Uppsala, Sweden). Null hypothesis for protein spots was rejected in case of ANOVA p<0.01, including false recovery rate correction (q<0.01). If not otherwise stated all chemicals were of analytical grade and obtained from GE Healthcare, Uppsala, Sweden.

### Proteome analysis of ZSB treated and untreated *P. aeruginosa* PAO1

Changes in the proteome were compared between a ZSB treated and an untreated *P. aeruginosa* PAO1, to acquire additional information on the mode of action of ZSB, and to verify the results of the proteomic comparison of the adapted and the non-adapted strain. Eight cultures in baffled Erlenmeyer flasks were prepared from four overnight pre-cultures by adding 1% of the final volume of pre-culture to 20 mL LB medium and grown for three hours to mid-log phase at 37°C. Then 0.1 mM (final concentration) of ZSB (solved in DMSO with a final concentration of 2% v/v) was added to four cultures and DMSO only (2% v/v final concentration) to the remaining four cultures (control group). The cultures were incubated for a further 3.5 h. A 4 mL sample was taken from each culture. The cells were harvested by centrifugation at 5000 *g* for 10 min and at room temperature (RT). The cell pellets were washed three times with 40 mM Tris (Carl Roth, Karlsruhe, Germany) buffer (pH 7.5) and stored at −80°C.

In order to optimise the sample preparation for 2-D DIGE the following changes in crude extract preparation were introduced in comparison to the proteome analysis of adapted and non-adapted strains. After thawing of the bacterial pellets (preparation as described above), 100 µL of 1% (v/v) sodium dodecyl sulphate (SDS) (GE Healthcare, Uppsala, Sweden) was added and incubated at 95°C for 5 minutes on a heating block (Thermomixer comfort, Eppendorf AG, Hamburg, Germany). Samples were subsequently cooled on ice for 5 min, and 250 µL of lysis buffer lacking protease inhibitor was added. To facilitate dissolving of proteins in the lysis buffer, samples were incubated under intermediate mixing for 1 h at room temperature prior to centrifugation. Labelling and electrophoretic separation of the first and second dimension was performed as described above. Due to significant changes in the proteome, only the protein spots with the highest difference in abundance (ANOVA p<0.01; false recovery rate correction q<0.01; standard log abundance ratio >2.5) were chosen for further analysis by means of mass spectrometry. Significantly differentially abundant protein spots were manually picked from preparative 2-DE gels that were prepared in parallel to 2-D DIGE analysis. Preparative 2-DE gels were stained by SyproRuby™ (Life Technologies GmbH, Darmstadt, Germany) according to manufacturers instructions.

Stained protein spots were excised from the gel and washed three times with acetonitrile/water (1∶1). The gel pieces were shrunk with acetonitrile, rehydrated in 50 mM NH_4_HCO_3_ and dried in a speedVac. The dried gel pieces were covered with aqueous solution containing 10 mM DTT and 50 mM NH_4_HCO_3._ The gel-embedded proteins were then reduced at 56°C for 45 min. Prior to in-gel digestion, the gel pieces were washed and dried, as previously described, to remove the reduction agent DTT. The gel pieces were then rehydrated in an ice-cold solution containing 10 ng µL^−1^ trypsin (sequencing grade, Promega, USA) and 10 mM NH_4_HCO_3_. After 45 min on ice, excessive trypsin solution was replaced by 20 µL of trypsin free buffer. Samples were stored over night at 37°C to ensure complete digestion of proteins. The digestion was arrested by addition of 20 µl 10% formic acid. The resulting peptides were extracted for 30 min at 37°C and further analyzed using ultra high performance liquid chromatography-mass spectrometry (UHPLC/MS).

UHPLC/MS data were acquired on an HCT ETD II iontrap mass spectrometer (Bruker Daltoniks, Bremen, Germany) equipped with a nano-ESI source (Bruker Daltonics, Bremen, Germany). Samples were introduced by an Easy-nLC 1000 system (Proxeon, Odense, Denmark) using a vented column setup comprising an 100 µm inner diameter (ID), 20 mm length (L) trapping column and an ID 75 µm, L 100 mm analytical column, both self packed with ReproSil-Pur C_18_-AQ, 5 µm (Dr. Maisch, Ammerbuch, Germany). A 5 to 18 µL sample was aspirated into the 25 µL sample loop and loaded onto the trap column using a flow rate of 6 µL min^−1^. Mobile phase solvent A: aqueous 0.1% formic acid; and solvent B: acetonitril were mixed with a linear gradient of 0% to 35% acetonitrile over 20 min with a flow rate of 300 nL min^−1^. The acetonitrile ratio was subsequently increased to 100% over 2 min and the column was regenerated in 100% ACN for an additional 8 min.

The Compass 3.0 software controlled data-dependent acquisition of MS and tandem MS (MS/MS) spectra. MS1 scans were acquired in standard enhanced mode. Five single scans in the mass range from m/z 400 to m/z 1400 were combined for one survey scan. Up to three doubly and triply charged ions rising above a given threshold were selected for MS/MS measurements. Ultrascan mode was used for the acquisition of MS2 data in the mass range from m/z 100 to m/z 1600, and three single scans were summed up. The ion charge control value was set to 250000 for all scan types. Peaklists in mascot generic format (mgf) were generated from the raw data by means of the Compass Data Analysis software module (Bruker Daltoniks, Bremen, Germany).

Proteins were identified by searching the NCBInr release 20090531 (National Center for Biotechnology Information, Bethesda, USA) using a local installation of MASCOT 2.2 (Matrix Science Ltd, London, UK). Searches were submitted via Proteinscape 2.1 (Bruker Daltoniks, Bremen, Germany) with the following parameter settings: Enzyme “trypsin”, species “eubacteria”, fixed modifications “carbamidomethyl”, optional modifications “methionine oxidation” and missed cleavages “2”. The mass tolerance was set to 0.4 Da for peptide and fragment spectra.

### Bacterial susceptibility tests of adapted *P. aeruginosa* PAO1

Comparative analysis of hydrogen peroxide tolerance and antibiotic susceptibility of adapted *P. aeruginosa* PAO1 was performed according to a modified protocol of The British Society for Antimicrobial Chemotherapy (BASC) for standardized disc susceptible testing (agar disc diffusion method) [29]. Liquid bacterial cultures of both the ZSB adapted and wildtype *P. aeruginosa* PAO1 were incubated for 18 h at 37°C. The cultures were diluted 1∶1 with sterile ultra pure water and OD_600_ was measured. To equalise the starting viable cell numbers, cultures with OD_600_ >0.3 were diluted 1∶1000 with ultra pure water and cultures with OD_600_ <0.3 were diluted 1∶500 to a final volume of 5 mL. The prepared cultures were then spread out onto Mueller-Hinton II agar plates (Sigma-Aldrich Chemie GmbH, Taufkirchen, Germany). For hydrogen peroxide tolerance, sterile filter paper disks, diameter = 10 mm (Sigma-Aldrich Chemie GmbH, Taufkirchen, Germany) impregnated with 15 µL of 30% hydrogen peroxide (Carl Roth, Karlsruhe, Germany), were added on top of the Mueller-Hinton II agar. Imipenem (10 µg) test discs (Oxoid, Basingstoke, UK) were used for antibacterial susceptibility tests. Cultures were incubated for 18–22 h at 37°C. After incubation the total zone of inhibition diameters (containing the filter paper disks) were determined with a sliding calliper. Measurements on tolerance against hydrogen peroxide were performed over seven days. Measurements on antibiotic susceptibility were performed on three different days. OriginPro 8.1 (OriginLab Corporation Northampton, UK) was used for statistic analysis, and the hypothesis was proven using the Mann-Whitney- Test.

### Degradation of ZSB by the ZSB adapted and the non-adapted P. *aeruginosa* PAO1

ZSB-degradation measurements are based on the work of Jacobson et al. [30]. Twenty millilitres of LB medium containing ZSB were inoculated with the ZSB- adapted (P_45_) and wildtype P. *aeruginosa* PAO1 (P_0_), and cultivated at 170 rpm for 18 h at 37°C. The concentration of ZSB was 1.3 µM for P_45_ and 0.5 µM for P_0_. After incubation the cultures were harvested by centrifugation at 5000 *g* for 10 min at RT and washed twice with ZSB-free LB medium. The OD_600_ of all samples was adjusted to 1.7 and the initial concentration of ZSB was set for all samples to 1.8 mM. 50 µL samples were taken at 30 min intervals for high performance liquid chromatography (HPLC) measurements. To monitor changes in the growth velocity, optical density measurements were performed every 60 min. After 5 h of incubation a viable cell count was performed. Samples for HPLC measurements were stored at −80°C until use. For the HPLC measurements the samples were diluted in ultra pure water for appropriate resolution. The chromatography was performed using an HPLC Agilent 1100 system (Agilent technologies, Santa Clara, USA). An aqueous solution of 60% 1 mM H_2_SO_4_ (Carl Roth, Karlsruhe, Germany) and 40% acetonitrile (Carl Roth, Karlsruhe, Germany) was used as an isocratic mobile phase at a flow rate of 1.2 mL min^−1^, the stationary phase was a L 250 mm x ID 4 mm EC Nucleodur 100-5 C_8_ ec (100 Å, 5 µM) column (Macherey-Nagel GmbH & Co. KG, Düren, Germany). The column temperature was set to 40°C. Sample injection volume was 20 µL. The HPLC was equipped with an Agilent diode array detector (DAD, λ 195–300 nm). The retention time of ZSB was 5.6 min. The measurement was determined at λ 220 nm, quantification was done on a calibration curve using the following ZSB concentrations: 0.05 mM, 0.2 mM, 0.5 mM, 0.75 mM, 1 mM, 1.5 mM, and 2 mM. Each experimental group consisted of four biological replicates.

In addition, the zinc uptake from the medium was measured. *P. aeruginosa* P_0_ and P_45_ were grown for 24 h in ZSB containing medium. The cultures were then centrifuged and the zinc content in the medium was measured by Atomic absorption spectroscopy (Atomic Absorption Spectrophotometer, Model 3030, Perkin-Elmer, Waltham, USA).

## Results

### Proteome analysis of ZSB treated *P. aeruginosa* PAO1

**Figure 1:**
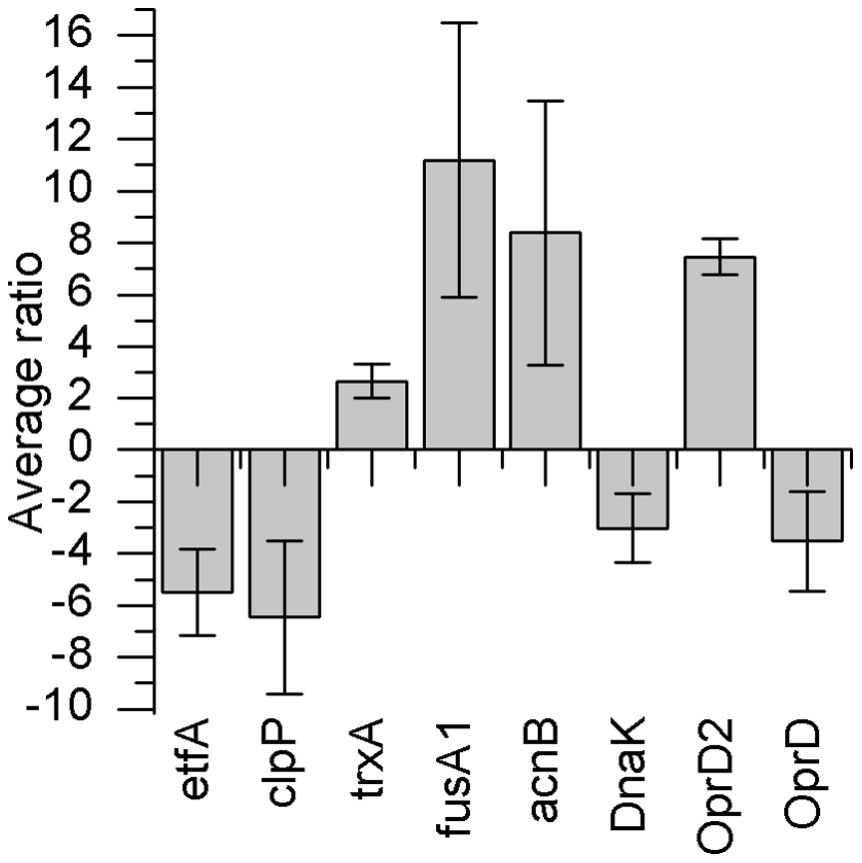
Average ratios of significantly different expressed proteins in comparative analysis of *P. aeruginosa* PAO1 treated with ZSB. 2-D DIGE proteome analysis was performed of non-adapted ZSB treated and untreated P_0_ strains. The following proteins exhibiting significantly different abundance where identified: etfA: electron transfer flavoprotein subunit alpha; clpP: ATP-dependent Clp protease proteolytic subunit; trxA: thioredoxin; fusA1: elongation factor G; acnB: bifunctional aconitate hydratase 2/2-methylisocitrate dehydratase; DnaK: molecular chaperone DnaK; OprD2: OprD2-like porin precursor; OprD: Chain A, crystal structure of the outer membrane protein OprD. Average ratio indicates the standardised volume ratio between two protein spots in two experimental populations [54].

In order to determine the influence of ZSB on the proteome of *P. aeruginosa*, the bacteria were treated with a relatively low concentration (0.12 mM) of ZSB. 2-D DIGE analysis yielded 647 significantly (ANOVA p<0.01, including false discovery rate (FDR) correction q<0.01) differently expressed protein spots, out of which 270 were down-regulated and 377 were up-regulated. From these protein spots, eight of the most significant differentially abundant proteins were identified using mass spectrometry. Protein spots that were identified could be defined as outer membrane proteins or their precursors. Different enzymes responsible for redox potential regulation were identified, as well as a chaperone and an electron transport chain protein, (see figures A to I in File S1). Figure 1 illustrates the mean protein abundance ratios (average ratios) of the ZSB treated *P. aeruginosa* PAO1 in relation to the untreated *P. aeruginosa* PAO1.

Protein abundances varied maximally from 11-times higher to approximately 7-times lower in the case of the ZSB treated *P. aeruginosa* PAO1. From the identified proteins, the following are connected to oxidative stress: thioredoxin (Accession: gi|15600433) [31], [32], elongation factor G (Accession: gi|15599462) [33], [34], bifunctional aconitate hydratase 2/2-methylisocitrate dehydratase (Accession: gi|15596984) [24], [50] ATP-dependent Clp protease proteolytic subunit (Accession: gi|15596998) [35], molecular chaperone DnaK (Accession: gi|15599955) [33], [36] (see figures A to I in File S1). In addition, one outer-membrane protein (OprD2-like porin precursor (Accession: gi|2645844)) was found to be up-regulated, whereas another related protein (outer membrane protein OprD (Accession: gi|158429225)) was found to be down-regulated.

### Microevolution of *P. aeruginosa* PAO1 to zinc Schiff-base

**Figure 2:**
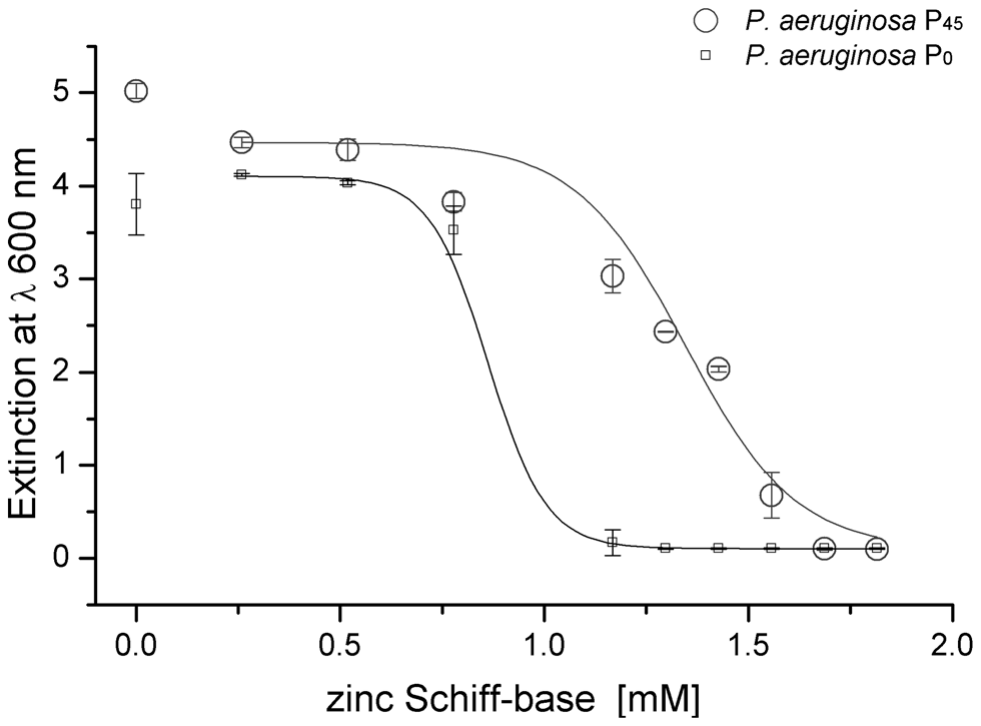
Comparison of growth of the ZSB adapted (P_45_) and the non-adapted (P_0_) *P. aeruginosa* PAO1. LB medium containing 2% (v/v) DMSO was used as control. A non-linear fit algorithm by dose-response function with variable Hill slope was used to fit both curves.

In order to define the starting point for microevolution experiments, the minimal inhibitory concentration of ZSB was determined for *P. aeruginosa* PAO1 in LB medium (figure 2). For microevolution a concentration of 0.5 mM ZSB was used as an initial concentration. The concentration of ZSB was then successively increased by 0.2 mM, or 0.4 mM, steps to 1.3 mM ZSB over a period of 45 passages. After each increase of the ZSB concentration, the optical density of the culture sharply decreased and subsequently increased in a gradual manner during the succeeding passages (see File S2).

The minimum inhibitory concentration of ZSB of the ZSB adapted (P_45_) and non-adapted (P_0_) *P. aeruginosa* PAO1 was determined. The results, depicted in figure 2, indicate that the MIC_50_ value of *P. aeruginosa* PAO1 P_45_ increased from 0.86 mM to 1.27 mM and the MIC_90_ from 1.03 mM to 1.6 mM, which corresponds to a 1.5 fold increase of MIC_50_ and 1.6 fold of MIC_90_.

To verify whether the increased tolerance against ZSB was as a result of increased metabolic activity or due to a heritable mutation, the adapted *P. aeruginosa* PAO1 P_45_ was passaged two times in ZSB free LB medium (P_45_L). The growth behaviour of P_45_L, as well as of the adapted *P. aeruginosa* PAO1 P_45_ and non-adapted P_0_ strain, were compared in LB medium containing 1.3 mM of ZSB. Growth (OD_600_ >0.5) was found for *P. aeruginosa* PAO1 P_45_ after less than 24 h, the P_45_L showed growth after 28 h. In the case of the *P. aeruginosa* PAO1 P_0_, no growth could be observed, even after 96 h of incubation.

### Proteomic analysis of the adapted and non-adapted *P. aeruginosa* PAO1

**Figure 3:**
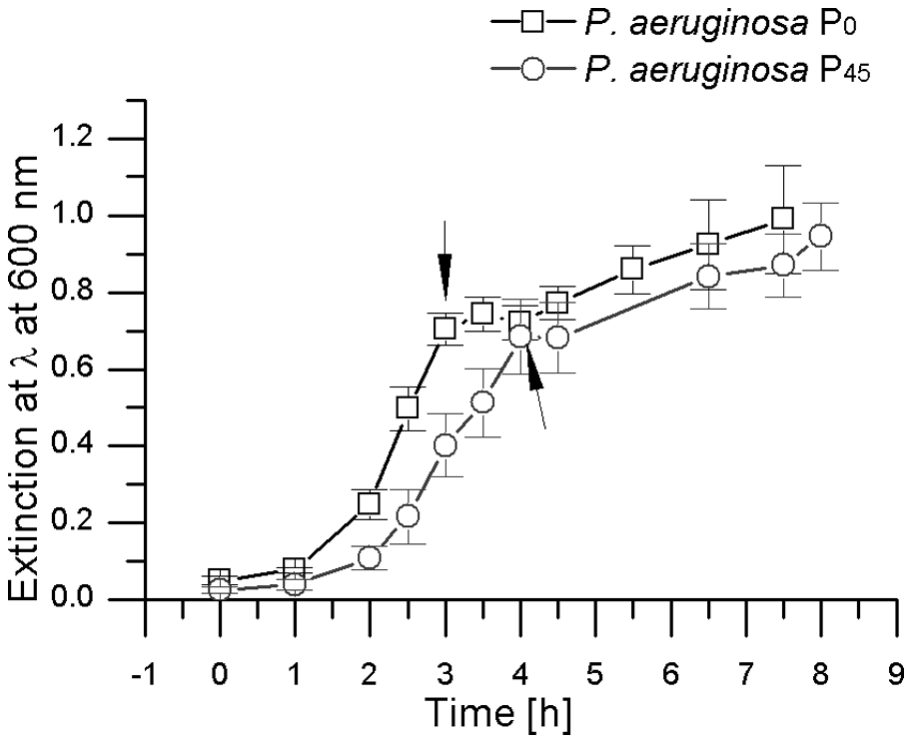
Growth curves of adapted P_45_ and non-adapted P_0_ *P. aeruginosa* PAO1. Black arrows indicate the addition of the ZSB. (n = 4).

In order to compare both *P. aeruginosa* PAO1 groups (P_0_ and P_45_) both strains were grown in the absence of ZSB to mid-log phase to an OD_600_ of 0.65. Then ZSB was added to both strains to a final concentration of 0 5 mM (addition indicated by arrows in figure 3). Both strains were grown in presence of ZSB for 3 h to 4 h at 37°C. As shown in figure 3 both cultures exhibit a similar growth pattern, with the exception that P_45_ exhibits a prolonged lag phase, most likely resulting from minor differences in the starting inoculum. Following addition of ZSB, growth of both cultures paused for a short time period, indicated by the plateau in figure 3.

**Figure 4:**
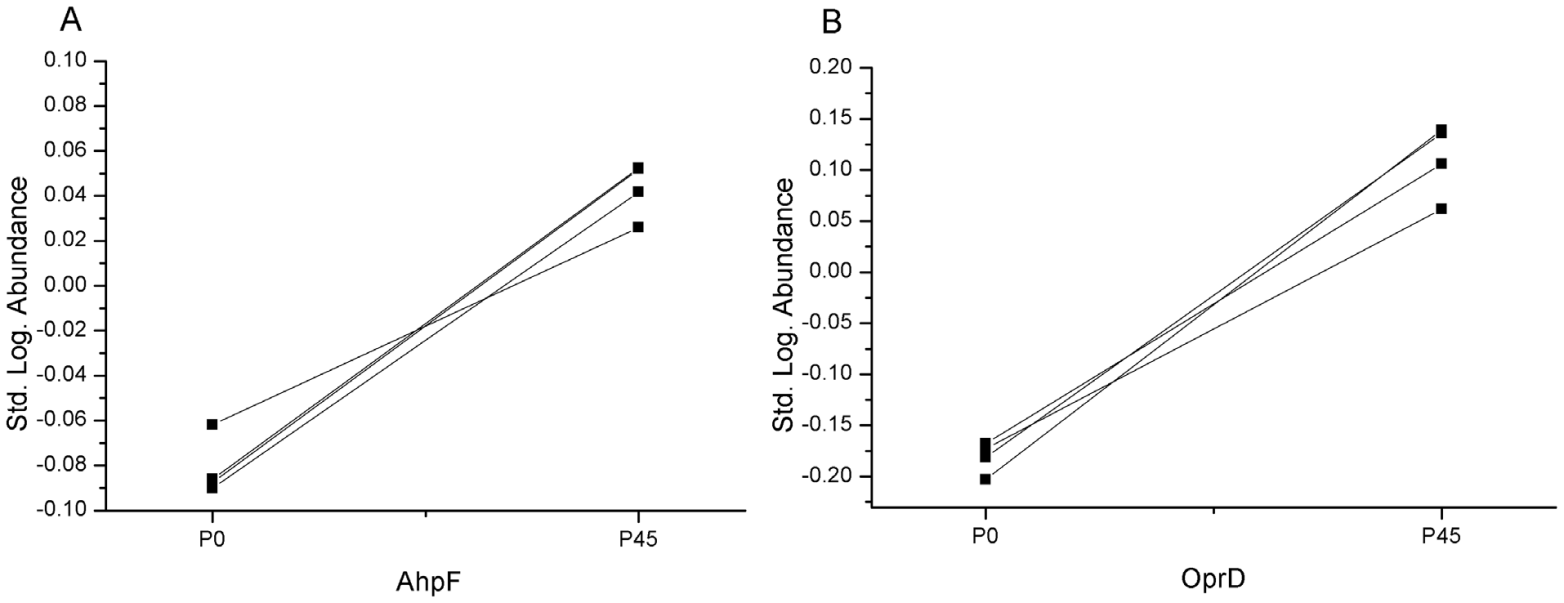
Logarithmic standard abundance of identified proteins of adapted and non-adapted *P. aeruginosa* PAO1. Logarithmic standard abundance is the decadal logarithm of the ratio of an individual protein spot and the internal standard within the same gel [54]. Individual values were in case of AhpF for P_0_ −0.087709; −0.086223; −0.06188; −0.090214 and P_45_ 0.051973; 0.05241; 0.025915; 0.041694. For OprD, the logarithmic abundances were in case of P_0_ −0.167843; −0.172957; −0.202919; −0.181265 and for P_45_ 0.105833; 0.0618; 0.139092; 0.136039. Exact ANOVA q-values (including FDR) for both protein spots were q = 0.008. Excluding FDR the p- value for AhpF was 1.33 10^−6^ and 5.7 10^−6^ for OprD respectively.

The proteomes of both the adapted and the non-adapted *P. aeruginosa* PAO1 were subsequently compared for changed protein expression by 2-D DIGE. Only two protein spots were found to be significantly different expressed (p<0.01). The proteins were identified by UHPLC-MS as alkyl hydroperoxide reductase subunit F (AhpF) from *P. aeruginosa* PAO1 (accession: gi|15595338), which was 1.3 times more abundant in the ZSB adapted strain, and the outer membrane protein OprD from *P. aeruginosa* (accession: gi|158429225), which was 2 times more abundant (see figures A to C in File S3). The logarithmic standard abundances of the identified proteins AhpF and OprD are shown in figure 4. As depicted in figure 4, AhpF and OprD are present in significantly lower abundances (q<0.01) in the unpassaged group than in the passaged P_45_ group.

### Bacterial susceptibility tests of adapted *P. aeruginosa* PAO1

Both 2-D DIGE experiments in combination with mass spectrometry of ZSB adapted (P_45_) and non-adapted (P_0_) *P. aeruginosa* PAO1 gave evidence of oxidative stress response when exposed to ZSB. Results of the comparative proteome analysis were therefore further verified by measuring the tolerance against the oxidative stressor hydrogen peroxide by agar diffusion test in triplicates on seven different days (n_total_  = 21). Table 1 shows the diameters of the individual zone of inhibition and the frequency of their occurrence.

**Table 1:** Inhibition zone diameters in relation to their frequency of occurrence in the experiment.

	P_0_					P_45_		
Diameter [mm]	56	54	52	50	48	52	50	48
n	1	1	6	11	2	3	11	7
Sum [n]	21					21		
Mean diameter [mm]	51					50		
Mann-Withney U	0.029*							

* Statistical significance using Student's T-Test was p = 0.018.

Using the Mann-Whitney-U-Test a p-value <0.05 was obtained. As indicated in table 1 a small but significant difference was found towards smaller inhibition zones for *P. aeruginosa* PAO1 P_45_. Hydrogen peroxide inhibited the growth of *P. aeruginosa* PAO1 P_45_ to a smaller extent than that of *P. aeruginosa* PAO1 P_0_. However, no significant change could be determined in the susceptibility against imipenem. Thus, the observed insensitivity against oxidative stress of P_45_ does not lead to imipenem resistance.

### Degradation of ZSB by the ZSB adapted and the non-adapted *P. aeruginosa* PAO1

**Figure 5:**
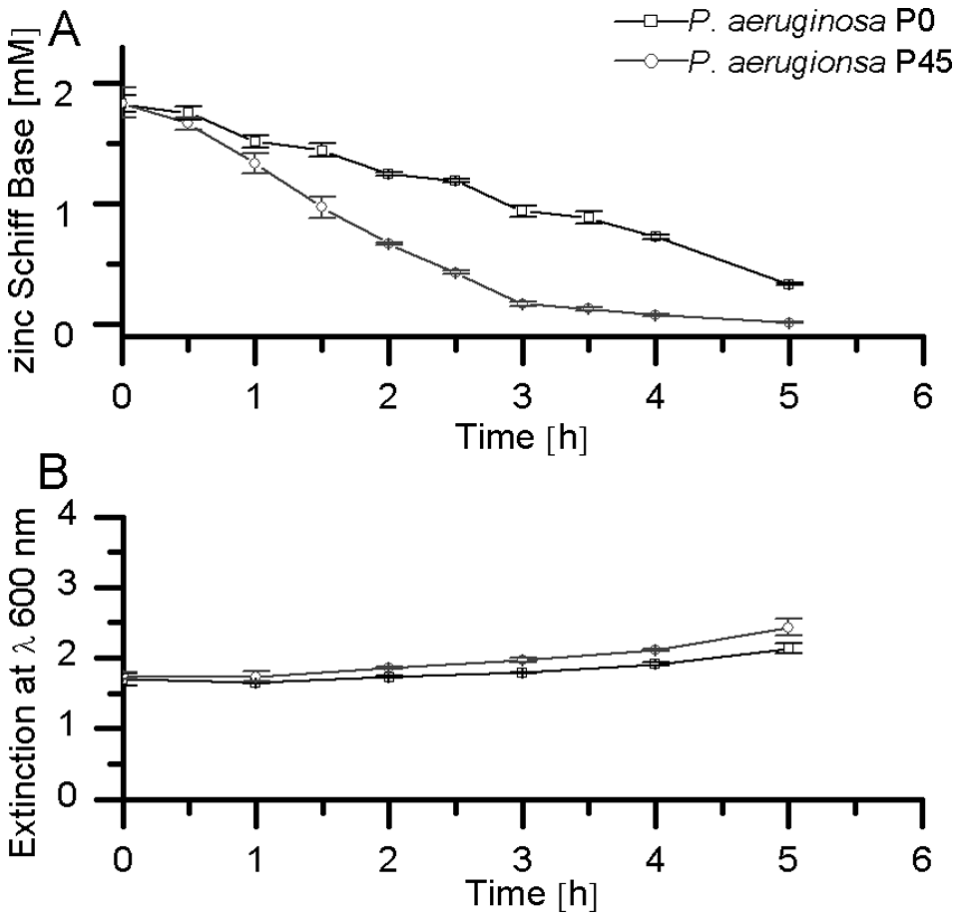
Degradation of ZSB by the adapted *P. aeruginosa* P_45_. (a) Degradation of ZSB by *P. aeruginosa* PAO1 P_0_ and *P. aeruginosa* PAO1 P_45_. (b) Optical density of *P. aeruginosa* P_45_ and P_0_ during incubation with ZSB. No difference (p>0.05) was found in the growth until the second hour of the experiment. Initial optical density for both experimental groups was 1.7, initial ZSB concentration was 1.8 mM. (n = 4).

An increased abundance of Ahp is known to be involved in facilitating the degradation and tolerance against aromatic compounds [30] [23] [24]. Figure 5 shows the ZSB concentration during incubation with *P. aeruginosa* PAO1 P_0_ and *P. aeruginosa* PAO1 P_45_. After five hours no ZSB could be found in case of the P_45_ cultures, whereas 0.3±0.1 mM could still be detected in the case of P_0_. After 24 h also for P_0_ the ZSB concentration fell below the analytical limit of detection (data not shown). As shown in figure 5 both strains (P_45_ and P_0_) were still able to grow in 1.8 mM ZSB, in a similar manner. Note that the initial cell density was much higher than that used in the experiment shown in figure 2, thus growth of both strains was still possible. No statistically significant difference (p>0.05) in bacterial growth could be determined until the second hour of incubation. In contrast, the actual ZSB concentration differed noticeably (p<0.05) between *P. aeruginosa* P_0_ and P_45_ after 30 minutes of incubation. After 1 h of incubation the ZSB concentrations differed by a factor of 1.1, and after 2 h by a factor of 1.9 (cP_0_/cP_45_). From a statistical point of view, the bacterial growth behaviour of both groups began to differ from the second hour on. The observed proportion of ZSB degradation at incubation times above 2 hours could have thus been additionally influenced by the slightly increased number of P_45_ in relation to P_0_ cells (OD600 of P_45_/OD600 of P_0_ after 5 h = 1.1).

Measurement of the zinc uptake by the bacteria from the medium did not show a significant difference for P_0_ and P_45_ (data not shown).

## Discussion

The ability of bacteria to rapidly adapt to environmental conditions is well documented, evidenced by the recent surge of antibiotic resistance exhibited by nosocomial pathogens. Various strategies are under investigation to stem the spread of resistant strains, most especially those antibiotic-resistant bacteria in hospitals. These strategies include implementation of strict hygiene sanctions, and incorporate the prospective use of surface disinfection by employing “auto-aseptic" surface coatings, such as zinc containing materials [37] [13]. The antimicrobial property of zinc is well known [38], as demonstrated by the zinc-containing material ZSB utilised by Poulter et al. for the deposition of antimicrobially-active coatings [15]. Due to the probable use of ZSB, or related materials, as antimicrobials, the development of resistances against such materials should be considered. For this reason *P. aeruginosa* PAO1 was recurrently incubated (passaged) for approximately 300 generations (45 passages) in ZSB, a material with known antibacterial properties [15]. Passaging yielded a MIC_50_ value increase by factor 1.6, showing adaptation. A study by Toprak et al. [39], who used a special device (“morbidostat”) for cultivation along with known biocides such as chloramphenicol, doxycycline and trimethoprim, demonstrated rising resistance in *Escherichia coli* within 20 days. In comparison to Lenski et al. [4], in our study it is likely that passaging of *P. aeruginosa* PAO1 for a higher number of generations could have led to an even greater tolerance against ZSB. Lenski et al. [4] showed, by comparing the fitness of minimal medium adapted *E. coli* in relation to non-adapted *E. coli*, that most of the adaptation occurs during the first 2000 generations. Molecular level mechanism descriptions of e.g. cell size were not mentioned in the latter study [4]. To gain further insight in to the mechanism of adaptation, and in to the mechanism of increased tolerance against ZSB, comparative studies of the proteome and physiology have been performed.

In our experiments the AhpF was found to be up-regulated in the ZSB adapted *P. aeruginosa* PAO1 P_45_. AhpF is expressed together with AhpC as a heterodimer forming the alkyl hydroperoxide reductase [23]. Ahp is known as a scavenger of hydrogen peroxides [40] and one function is the reduction of alkyl hydroperoxides to alcohols [30] [41]. Even AhpF alone can have advantageous effects on the resistance against reactive oxygen species (ROS) as shown by Poole and Ellis [41]. Kang et al. [24] reported that degradation of aromatic compounds such as naphthalene leads to the formation of reactive oxygen species. Moreover, the results of Kang et al [24] and Jacobson et. al [30] showed that over-expression of, amongst others, alkyl hydroperoxide reductase enhances the degradation of these compounds whilst increasing the tolerance to both naphthalene and ROS. This agrees with our results of the agar diffusion tests against hydrogen peroxide, where a small but significant increase in the tolerance was determined in the case of *P. aeruginosa* PAO1 P_45_. Moreover, *P. aeruginosa* PAO1 P_45_ degraded ZSB significantly faster than *P. aeruginosa* PAO1 P_0_. In accordance with the literature, our results suggest involvement of Ahp in the defence mechanism against ROS formed during the metabolic degradation of ZSB by the bacteria. The observed up-regulation of AhpF appears to be advantageous for the improved survivability of ZSB adapted *P. aeruginosa* PAO1 P_45_. The literature suggests that up-regulation of Ahp might also be involved in the observed accelerated degradation of ZSB by *P. aeruginosa* PAO1 P_45_.

Proteome analysis of the ZSB adapted *P. aeruginosa* PAO1 (P_45_) compared to wildtype resulted in an observed increase in the level of the outer membrane protein OprD. Huang and Hancock demonstrated that strong over-expression of OprD leads to elevated susceptibility against carbapenemes [42]. No statistically significant change (p = 0.08) in the susceptibility against the carbapeneme Imipenem could be observed in the case of P_45_. The development of resistance against ZSB does not necessarily result in the development of co-resistances. To gain deeper knowledge about the influence of ZSB on the proteome, and to verify the results of the proteome analysis of *P. aeruginosa* PAO1 P_0_ and P_45_, the non-adapted *P. aeruginosa* PAO1 P_0_ was treated with low concentrations of ZSB for a short period (3.5 h) , and compared to untreated P_0_. Although working at low concentrations, a dramatic change in the proteome was observed for ZSB treated *P. aeruginosa* PAO1 P_0_. Outer membrane proteins and enzymes connected to oxidative stress were found to be significantly differently expressed. Interestingly, the OprD was found to be down-regulated here. Ochs et al. [43] demonstrated that upon short-time co-incubation with acetylsalicylate, salicylate or benzoate, OprD is repressed. Our results are in good agreement with the latter, as the ZSB ligand SB is a salicylaldehyde [15], and thus possesses a similar structure. In addition, Conejo et al. [44] and Martinez-Martinez et al. [45] showed that upon addition of zinc (as zinc salt or zinc eluted from catheters) to the culture medium, OprD is also repressed and thus the tolerance for antibiotics of the carbapenem group is increased. This effect was found to be reversible [45].

Our data indicates that short-term exposure to ZSB leads to down-regulation of OprD expression, whereas after adaptation the expression of OprD is less affected by ZSB. It is our view that the changed regulation of OprD is not a result of adaptation to ZSB. It is more likely a result of the adaptation to the pure LB medium. This is supported by the fact that OprD is responsible for uptake of basic amino acids under physiological cell conditions [46] [43] [42]. In our study we observed an increase in the optical densities after six passages of *P. aeruginosa* PAO1 P_0_, after 24 h of growth in LB. A similar effect was observed in the work of Perron et al. [47] who performed microevolution experiments on an antimicrobial peptide.

The other group of proteins found in the comparative proteome analysis of the short exposure ZSB treated *P. aeruginosa* PAO1 P_0_ could be connected to oxidative stress. The thioredoxin, the bifunctional aconitate hydratase 2/2-methylisocitrate dehydratase, ATP-dependent Clp protease, elongation factor G and DnaK proteins were found to be differentially regulated.

Thioredoxin was found to be up-regulated. Thioredoxins, together with glutaredoxins, are the main groups of proteins responsible for maintaining the reducing potential of a cell [48]. Thioredoxin was found to play a crucial role in the oxidative stress response. Das and Das [32] have shown that thioredoxin on its own is able to scavenge reactive oxygen species, such as hydroxyl radicals. Moreover, mutant and adaptive response analyses have highlighted the importance of increased expression of thioredoxin in response to oxidative stress [49].

Our results have shown that bifunctional aconitate hydratase 2/2-methylisocitrate dehydratase (aconitase) was present in higher abundance in the proteome of the ZSB treated *P. aeruginosa* PAO1. Experiments on *E. coli* have shown that aconitase activity is decreased during oxidative stress [50]. In contrast, the abundance of the protein increases with increasing concentration of the oxidative agent [50].

DnaK together with ATP-dependent Clp protease are described as heat shock proteins. In our experiments both proteins were found to be down-regulated. Tamarit et al. [33] showed that DnaK is one of the major targets for oxidative stress by hydrogen peroxide in *E. coli,* DnaK is post-translational inactivated under oxidative stress conditions in favour of the chaperone Hsp33, which gets activated [36]. Salunkhe et al. [35] suggest as a reason for down-regulation of ATP-dependent Clp proteases to reduce the rate of protein degradation. In the same study it was stated that prolyl-peptidyl isomerases exhibited increased expression to counteract protein dysfunction by misfolding.

Our data revealed a substantial increase in the elongation factor G (EF-G). This protein has an essential function in protein biosynthesis with respect to translocation of the tRNA from the A to the P site of the ribosome [51]. During oxidative stress EF-G is found to be readily inactivated by oxidation in the carboxyl-terminal region [33]. Increased expression of EF-G to compensate for the inactivation during oxidative stress conditions would, therefore, be favourable to maintain translation. Stronger expression of RNAs encoding for ribosomal proteins was also found by Salunkhe et al [35].

One electron transfer flavoprotein was found to be less abundant in the proteome of ZSB treated *P. aeruginosa* PAO1. The electron transfer flavoproteines can be classified into two groups. In one group it is expressed continuously, as a house-keeping protein; where as in the second group it is only expressed under defined circumstances [52]. Lower abundance of this protein in presence of ZSB is not surprising, as the protein is involved in energy metabolism and the ZSB treated *P. aeruginosa* PAO1 exhibited delayed growth.

No change in expression of proteins connected to zinc homeostasis was found. In addition, no significant change in the Zn uptake from the medium could be determined.

In summary, proteomic analyses indicate protein expression profiles exhibited by cells undergoing oxidative stress. The stress is caused by the hydrocarbon component of the ZSB. It was demonstrated by an additional experiment on the organic compound of the Schiff-base alone that the bactericidal effect was remarkably reduced by the addition of zinc (see File S4). This effect may be associated with the fact that zinc at certain concentrations is recognized as an antioxidant [53].

According to the literature reactive oxygen species (ROS) occur during degradation of aromatic and aliphatic hydrocarbons [24]: namely the up-regulation of Ahp was reported to be part of the defence mechanism combating ROS [30] and in promoting accelerated degradation of toxic organic compounds [24] [23]. Therefore we assume that the increased ZSB tolerance of the passaged *P. aeruginosa* PAO1 P_45_ is apparently related to the increase of AhpF, or Ahp, respectively.

It is likely that AhpF up-regulation enables *P. aeruginosa* to reduce hydrocarbon peroxides, formed during degradation of the hydrocarbon part of the ZSB, significantly faster and thus provides an advantage in the growth rate in contrast to the non-adapted *P. aeruginosa* PAO1 P_0_.

## Conclusions

We were able to demonstrate adaptation of *P. aeruginosa* PAO1 against the ZSB (bis(N-allylsalicylideneiminato)-zinc) within a relatively short time period. Our results indicate that *P. aeruginosa* degrades ZSB, which leads to the formation of ROS. The adapted *P. aeruginosa* PAO1 displays a higher tolerance towards hydrogen peroxide and is able to degrade ZSB significantly faster. The slightly higher abundance of AhpF suggests involvement of alkyl hydroperoxide reductase in the defence mechanism against ROS and in the acquired ZSB tolerance. The accelerated degradation of toxic organic compounds has also been associated with up-regulation of Ahp in literature. With reference to the literature this gives some hint that Ahp might also be involved in the observed accelerated ZSB degradation by the ZSB adapted strain. Additional experiments with mutants possessing knocked-out or overexpressed Ahp may elucidate further the role of Ahp, both in the observed accelerated degradation of ZSB and in the defence mechanism against ROS. Further investigations are therefore needed to fully elucidate the underlying mechanism and should thus be part of future work.

In summary, the speed of adaptation of microbes is an additional, and arguably critical, factor that should be considered in the development of antimicrobial substances, as demonstrated in this study.

## Supporting Information

File S1 Single channel image of a two-dimensional difference gel and peptide mass fingerprints.
(DOC)

File S2 Time course of adaptation of *P. aeruginosa* PAO1 in LB medium with increasing concentrations of zinc Schiff-base.
(DOC)

File S3 Single channel image of a two-dimensional difference gel and peptide mass fingerprints.
(DOC)

File S4 Measurement of intracellular hydrogenperoxide formation using 2,7-dichlorodihydro-fluorescein diacetate.
(DOC)
