# Supplementary material for: Insights into Mechanisms and Proteomic Characterisation of Pseudomonas aeruginosa Adaptation to a Novel Antimicrobial Substance
Source: PLoS One. 2013 Jul 15;8(7):e66862. doi: 10.1371/journal.pone.0066862 (PMC3711899; doi:10.1371/journal.pone.0066862)
Supplement: File S1 — Single channel image of a two-dimensional difference gel and peptide mass fingerprints. (DOC) [file pone.0066862.s001.doc]

**1.1 Representative two-dimensional difference gel of ZSB of intoxicated and non-intoxicated *P. aeruginosa* PAO1**


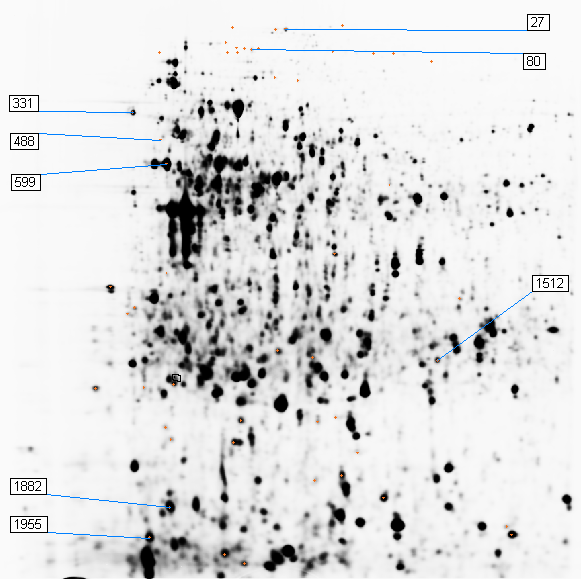


**Figure A. Single channel image of a two-dimensional difference gel with a pH range from pH 4-7, 18 cm.** Proteins of significantly different abundance (ANNOVA p < 0.01, q < 0.01) are marked with a red dot. The selected proteins are annotated with numbers.

1. 2 Detailed Protein Report proteins displaying significantly different abundance between ZSB intoxicated and non-intoxicated *P. aeruginosa* PAO1

Figures below show the peptide mass fingerprints (PMF) of protein spots highlighted in figure A

**Electron transfer flavoprotein alpha-subunit, Pseudomonas aeruginosa PAO1**

Accession: gi|15598147 Score: 109.3

Database: NCBInr(NCBInr_20101130.fasta) MW [kDa]: 31.4

Database Date: 2010-12-02 pI: 4.8

Modification(s): Oxidation Sequence Coverage [%]: 12.3

No. of unique Peptides: 3

**
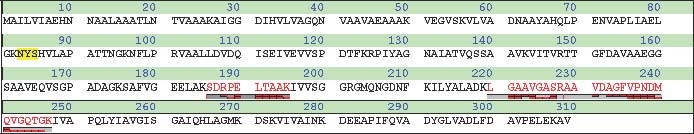
**

| **Cmpd.** | **No. of Cmpds.** | **m/z** | **Δ m/z [ppm]** | **z** | **Rt [min]** | **Score** | **P** | **Range** | **Sequence** | **Modification** |
| --- | --- | --- | --- | --- | --- | --- | --- | --- | --- | --- |
| **8** | 1 | 544.4000 | 200.73 | 2 | 10.3 | 31.6 | 1 | 186-195 | K.SDRPELTAAK.I |  |
| **5** | 1 | 401.3000 | 168.23 | 2 | 9.9 | 30.9 | 0 | 220-228 | K.LGAAVGASR.A |  |
| **144** | 1 | 961.5000 | 562.31 | 2 | 15.8 | 48.1 | 0 | 229-247 | R.AAVDAGFVPNDMQVGQTGK.I | Oxidation: 12 |

Figure B. Peptide mass fingerprint search result of the selected protein spot labelled in section 1.1 (**figure A)** with 1882

**ATP-dependent Clp protease proteolytic subunit, Pseudomonas aeruginosa PAO1**

Accession: gi|15596998 Score: 301.9

Database: NCBInr(NCBInr_20101130.fasta) MW [kDa]: 23.5

Database Date: 2010-12-02 pI: 6.0

Modification(s): Carbamidomethyl, Oxidation Sequence Coverage [%]: 32.4

No. of unique Peptides: 9

**
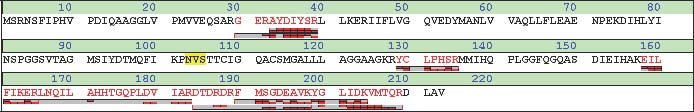
**

| **Cmpd.** | **No. of Cmpds.** | **m/z** | **Δ m/z [ppm]** | **z** | **Rt [min]** | **Score** | **P** | **Range** | **Sequence** | **Modification** |
| --- | --- | --- | --- | --- | --- | --- | --- | --- | --- | --- |
| **59** | 1 | 615.4000 | 163.98 | 2 | 13.2 | 29.7 | 1 | 30-39 | R.GERAYDIYSR.L |  |
| **73** | 1 | 444.3000 | 187.93 | 2 | 13.7 | 44.3 | 0 | 33-39 | R.AYDIYSR.L |  |
| **22** | 1 | 466.8000 | 162.87 | 2 | 11.6 | 36.9 | 0 | 129-135 | R.YCLPHSR.M | Carbamidomethyl: 2 |
| **206** | 1 | 524.4000 | 164.99 | 2 | 17.2 | 46.5 | 1 | 158-165 | K.EILFIKER.L |  |
| **259** | 1 | 500.1000 | 635.03 | 4 | 18.4 | 23.9 | 0 | 166-183 | R.LNQILAHHTGQPLDVIAR.D |  |
| **6** | 1 | 587.0000 | 687.32 | 3 | 11.0 | 20.3 | 2 | 184-198 | R.DTDRDRFMSGDEAVK.Y | Oxidation: 8 |
| **4** | 1 | 500.3000 | 147.48 | 2 | 10.1 | 32.9 | 0 | 190-198 | R.FMSGDEAVK.Y | Oxidation: 2 |
| **83** | 1 | 354.7000 | 0.08 | 2 | 14.0 | 37.6 | 0 | 199-204 | K.YGLIDK.V |  |
| **128** | 1 | 670.4000 | 66.29 | 2 | 15.2 | 29.8 | 1 | 199-209 | K.YGLIDKVMTQR.D | Oxidation: 8 |

Figure C. Peptide mass fingerprint search result of the selected protein spot labelled in section 1.1 (**figure A)** with 1512.

**Thioredoxin, Pseudomonas aeruginosa PAO1**

Accession: gi|15600433 Score: 243.6

Database: NCBInr(NCBInr_20101130.fasta) MW [kDa]: 11.9

Database Date: 2010-12-02 pI: 4.5

Modification(s): Oxidation Sequence Coverage [%]: 46.3

No. of unique Peptides: 5

**
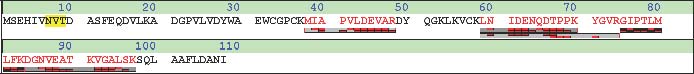
**

| **Cmpd.** | **No. of Cmpds.** | **m/z** | **Δ m/z [ppm]** | **z** | **Rt [min]** | **Score** | **P** | **Range** | **Sequence** | **Modification** |
| --- | --- | --- | --- | --- | --- | --- | --- | --- | --- | --- |
| **185** | 1 | 615.4000 | 111.23 | 2 | 18.3 | 53.0 | 0 | 38-48 | K.MIAPVLDEVAR.D | Oxidation: 1 |
| **24** | 1 | 692.4000 | 84.98 | 2 | 12.9 | 73.1 | 0 | 59-70 | K.LNIDENQDTPPK.Y |  |
| **67** | 1 | 620.7000 | 621.23 | 3 | 14.7 | 52.1 | 1 | 59-74 | K.LNIDENQDTPPKYGVR.G |  |
| **353** | 1 | 518.4000 | 194.88 | 2 | 22.5 | 28.2 | 0 | 75-83 | R.GIPTLMLFK.D | Oxidation: 6 |
| **15** | 1 | 694.9000 | 39.39 | 2 | 12.6 | 37.1 | 1 | 84-97 | K.DGNVEATKVGALSK.S |  |

Figure D. Peptide mass fingerprint search result of the selected protein spot labelled in section 1.1 (**figure A)** with 1955.

**Elongation factor G, Pseudomonas aeruginosa PAO1**

Accession: gi|15599462 Score: 575.5

Database: NCBInr(NCBInr_20101130.fasta) MW [kDa]: 77.7

Database Date: 2010-12-02 pI: 4.9

Modification(s): Carbamidomethyl, Oxidation Sequence Coverage [%]: 20.3

No. of unique Peptides: 12

**
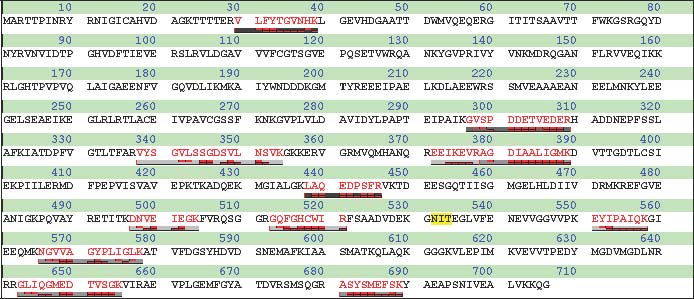
**

| **Cmpd.** | **No. of Cmpds.** | **m/z** | **Δ m/z [ppm]** | **z** | **Rt [min]** | **Score** | **P** | **Range** | **Sequence** | **Modification** |
| --- | --- | --- | --- | --- | --- | --- | --- | --- | --- | --- |
| **106** | 1 | 589.4000 | 132.61 | 2 | 16.4 | 36.2 | 0 | 30-39 | R.VLFYTGVNHK.L |  |
| **15** | 1 | 724.4000 | 120.39 | 2 | 12.5 | 65.9 | 0 | 297-309 | K.GVSPDDETVEDER.H |  |
| **249** | 1 | 855.9000 | -57.32 | 2 | 20.2 | 27.1 | 0 | 338-354 | R.VYSGVLSSGDSVLNSVK.G |  |
| **1** | 1 | 451.8000 | 109.08 | 2 | 9.8 | 28.6 | 1 | 372-378 | R.EEIKEVR.A |  |
| **209** | 1 | 538.3000 | 10.33 | 2 | 19.1 | 72.8 | 0 | 379-389 | R.AGDIAALIGMK.D | Oxidation: 10 |
| **39** | 1 | 531.8000 | 67.02 | 2 | 13.7 | 71.2 | 0 | 438-446 | K.LAQEDPSFR.V |  |
| **7** | 1 | 452.3000 | 166.87 | 2 | 11.1 | 22.3 | 0 | 497-504 | K.DNVEIEGK.F |  |
| **141** | 1 | 580.8000 | 43.83 | 2 | 17.1 | 40.8 | 0 | 513-521 | R.GQFGHCWIR.F | Carbamidomethyl: 6 |
| **87** | 1 | 481.3000 | 59.65 | 2 | 15.6 | 43.4 | 0 | 551-558 | K.EYIPAIQK.G |  |
| **291** | 1 | 651.0000 | 176.97 | 2 | 21.4 | 45.7 | 0 | 566-578 | K.NGVVAGYPLIGLK.A |  |
| **44** | 1 | 675.9000 | 100.44 | 2 | 14.0 | 53.5 | 0 | 643-655 | R.GLIQGMEDTVSGK.V | Oxidation: 6 |
| **96** | 1 | 525.3000 | 125.56 | 2 | 16.0 | 35.8 | 0 | 681-689 | R.ASYSMEFSK.Y |  |

Figure E. Peptide mass fingerprint search result of the selected protein spot labelled in section 1.1 (**figure A)** with 80.

**Bifunctional aconitate hydratase 2/2-methylisocitrate dehydratase, Pseudomonas aeruginosa PAO1**

Accession: gi|15596984 Score: 726.2

Database: NCBInr(NCBInr_20090531.fasta) MW [kDa]: 93.6

Database Date: 2009-06-10 pI: 5.1

Modification(s): Carbamidomethyl, Oxidation Sequence Coverage [%]: 19.7

No. of unique Peptides: 17

**
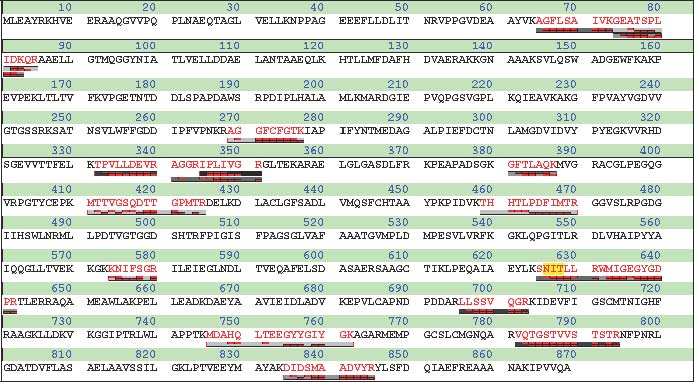
**

| **Cmpd.** | **No. of Cmpds.** | **m/z** | **Δ m/z [ppm]** | **z** | **Rt [min]** | **Score** | **P** | **Range** | **Sequence** | **Modification** |
| --- | --- | --- | --- | --- | --- | --- | --- | --- | --- | --- |
| **121** | 1 | 370.3000 | 171.52 | 3 | 17.0 | 29.5 | 1 | 341-351 | R.AGGRIPLIVGR.G |  |
| **136** | 1 | 384.3000 | 102.71 | 2 | 17.4 | 37.5 | 0 | 345-351 | R.IPLIVGR.G |  |
| **49** | 1 | 382.8000 | 212.44 | 2 | 14.7 | 20.3 | 0 | 381-387 | K.GFTLAQK.M |  |
| **3** | 1 | 807.9000 | 51.32 | 2 | 10.7 | 30.2 | 0 | 411-425 | K.MTTVGSQDTTGPMTR.D | Oxidation: 1, 13 |
| **164** | 1 | 495.6000 | 36.31 | 3 | 18.2 | 28.0 | 0 | 459-470 | K.THHTLPDFIMTR.G | Oxidation: 10 |
| **32** | 1 | 411.3000 | 157.95 | 2 | 13.6 | 40.0 | 1 | 574-580 | K.KNIFSGR.I |  |
| **106** | 1 | 408.8000 | 121.03 | 2 | 16.6 | 28.7 | 0 | 625-631 | K.SNITLLR.W |  |
| **165** | 1 | 648.8000 | 19.25 | 2 | 18.1 | 51.8 | 0 | 632-642 | R.WMIGEGYGDPR.T | Oxidation: 2 |
| **13** | 1 | 430.3000 | 108.23 | 2 | 12.2 | 39.3 | 0 | 696-703 | R.LLSSVQGR.K |  |
| **146** | 1 | 664.4000 | 150.70 | 3 | 17.7 | 30.1 | 0 | 746-762 | K.MDAHQLTEEGYYGIYGK.A | Oxidation: 1 |
| **6** | 1 | 661.9000 | 76.82 | 2 | 11.8 | 104.3 | 0 | 782-794 | R.VQTGSTVVSTSTR.N |  |
| **78** | 1 | 636.3000 | 27.88 | 2 | 15.7 | 39.9 | 0 | 835-845 | K.DIDSMAADVYR.Y | Oxidation: 5 |

Figure F. Peptide mass fingerprint search result of the selected protein spot labelled in section 1.1 (**figure A)** with 27.

**Molecular chaperone DnaK, Pseudomonas aeruginosa PAO1**

Accession: gi|15599955 Score: 480.0

Database: NCBInr(NCBInr_20090531.fasta) MW [kDa]: 68.4

Database Date: 2009-06-10 pI: 4.6

Modification(s): Oxidation Sequence Coverage [%]: 22.8

No. of unique Peptides: 12

**
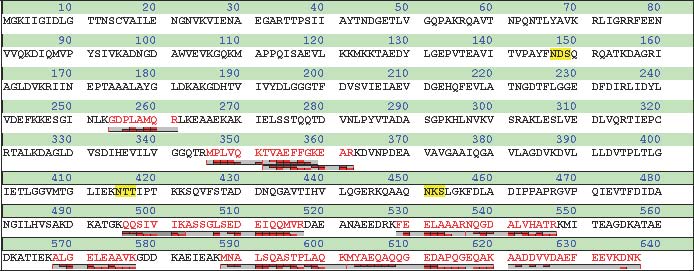
**

| **Cmpd.** | **No. of Cmpds.** | **m/z** | **Δ m/z [ppm]** | **z** | **Rt [min]** | **Score** | **P** | **Range** | **Sequence** | **Modification** |
| --- | --- | --- | --- | --- | --- | --- | --- | --- | --- | --- |
| **11** | 1 | 452.3000 | 174.08 | 2 | 11.5 | 33.4 | 0 | 254-261 | K.GDPLAMQR.L | Oxidation: 6 |
| **4** | 1 | 366.2000 | -26.35 | 2 | 9.8 | 26.5 | 0 | 346-351 | R.MPLVQK.T | Oxidation: 1 |
| **164** | 1 | 449.8000 | 139.89 | 2 | 18.6 | 34.7 | 0 | 352-359 | K.TVAEFFGK.E |  |
| **136** | 1 | 627.9000 | 115.49 | 2 | 17.4 | 31.9 | 1 | 352-362 | K.TVAEFFGKEAR.K |  |
| **32** | 1 | 408.3000 | 115.36 | 2 | 13.3 | 41.0 | 0 | 496-502 | K.QQSIVIK.A |  |
| **80** | 1 | 833.4000 | 10.73 | 2 | 15.7 | 36.6 | 0 | 503-517 | K.ASSGLSEDEIQQMVR.D | Oxidation: 13 |
| **49** | 1 | 453.8000 | 137.49 | 2 | 14.6 | 34.0 | 0 | 529-536 | K.FEELAAAR.N |  |
| **13** | 1 | 591.4000 | 161.14 | 2 | 11.6 | 56.4 | 0 | 537-547 | R.NQGDALVHATR.K |  |
| **120** | 1 | 500.8000 | 25.35 | 2 | 17.1 | 32.1 | 0 | 568-577 | K.ALGELEAAVK.G |  |
| **52** | 1 | 738.4000 | 27.40 | 2 | 14.7 | 56.1 | 0 | 588-601 | K.MNALSQASTPLAQK.M | Oxidation: 1 |
| **10** | 1 | 699.3000 | 468.38 | 3 | 11.3 | 59.1 | 0 | 602-620 | K.MYAEQAQQGEDAPQGEQAK.A | Oxidation: 1 |
| **163** | 1 | 632.0000 | 60.24 | 3 | 18.5 | 22.1 | 1 | 621-637 | K.AADDVVDAEFEEVKDNK. |  |

Figure G. Peptide mass fingerprint search result of the selected protein spot labelled in section 1.1 (**figure A)** with 331.

**OprD2-like porin precursor, Pseudomonas aeruginosa PAO1**

Accession: gi|2645844 Score: 475.6

Database: NCBInr(NCBInr_20101130.fasta) MW [kDa]: 49.7

Database Date: 2010-12-02 pI: 4.8

Sequence Coverage [%]: 16.1

No. of unique Peptides: 7

**
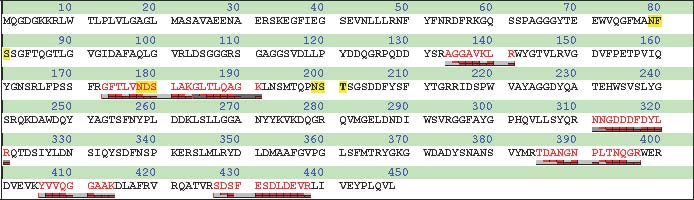
**

| **Cmpd.** | **No. of Cmpds.** | **m/z** | **Δ m/z [ppm]** | **z** | **Rt [min]** | **Score** | **P** | **Range** | **Sequence** | **Modification** |
| --- | --- | --- | --- | --- | --- | --- | --- | --- | --- | --- |
| **1** | 1 | 386.3000 | 141.33 | 2 | 9.9 | 45.0 | 1 | 134-141 | R.AGGAVKLR.W |  |
| **169** | 1 | 582.9000 | 143.11 | 2 | 19.1 | 107.2 | 0 | 173-183 | R.GFTLVNDSLAK.G |  |
| **21** | 1 | 394.3000 | 159.16 | 2 | 13.5 | 45.0 | 0 | 184-191 | K.GLTLQAGK.L |  |
| **147** | 2 | 672.3000 | 31.89 | 2 | 18.3 | 92.9 | 0 | 311-321 | R.NNGDDDFDYLR.Q |  |
| **5** | 1 | 679.4000 | 108.37 | 2 | 12.6 | 67.1 | 0 | 385-397 | R.TDANGNPLTNQGR.W |  |
| **4** | 1 | 446.8000 | 116.43 | 2 | 10.3 | 42.6 | 0 | 406-414 | K.YVVQGGAAK.D |  |
| **149** | 1 | 699.8000 | -6.59 | 2 | 18.4 | 77.1 | 0 | 427-438 | R.SDSFESDLDEVR.L |  |

Figure H. Peptide mass fingerprint search result of the selected protein spot labelled in section 1.1 (**figure A)** with 488.

**Chain A, crystal structure of the outer membrane protein Oprd, Pseudomonas aeruginosa**

Accession: gi|158429225 Score: 930.0

Database: NCBInr(NCBInr_20091130.fasta) MW [kDa]: 47.0

Database Date: 2009-12-04 pI: 5.3

Modification(s): Oxidation Sequence Coverage [%]: 31.3

No. of unique Peptides: 17

**
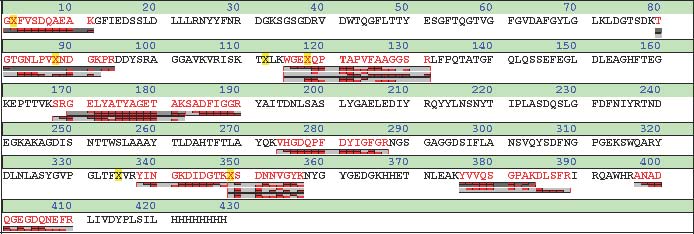
**

| **Cmpd.** | **No. of Cmpds.** | **m/z** | **Δ m/z [ppm]** | **z** | **Rt [min]** | **Score** | **P** | **Range** | **Sequence** | **Modification** |
| --- | --- | --- | --- | --- | --- | --- | --- | --- | --- | --- |
| **72** | 3 | 590.8000 | 37.80 | 2 | 13.0 | 66.5 | 0 | 1-11 | -.GEFVSDQAEAK.G |  |
| **39** | 1 | 660.3000 | -25.19 | 2 | 11.9 | 40.3 | 0 | 80-92 | K.TGTGNLPVMNDGK.P | Oxidation: 9 |
| **23** | 1 | 525.0000 | 109.58 | 3 | 11.3 | 53.8 | 1 | 80-94 | K.TGTGNLPVFNDGKPR.D |  |
| **295** | 1 | 881.4000 | -25.52 | 2 | 19.8 | 30.8 | 0 | 115-131 | K.WGEMQPTAPVFAAGGSR.L |  |
| **232** | 1 | 889.4000 | -22.43 | 2 | 18.1 | 98.8 | 0 | 115-131 | K.WGEMQPTAPVFAAGGSR.L | Oxidation: 4 |
| **233** | 1 | 593.3000 | 11.13 | 3 | 18.1 | 51.0 | 0 | 115-131 | K.WGEFQPTAPVFAAGGSR.L |  |
| **124** | 1 | 808.9000 | 0.77 | 2 | 14.9 | 77.2 | 1 | 168-182 | K.SRGELYATYAGETAK.S |  |
| **150** | 3 | 687.3000 | -47.73 | 2 | 16.1 | 104.9 | 0 | 170-182 | R.GELYATYAGETAK.S |  |
| **102** | 1 | 411.7000 | -21.52 | 2 | 14.3 | 48.7 | 0 | 183-190 | K.SADFIGGR.Y |  |
| **333** | 1 | 804.4000 | 20.27 | 2 | 20.8 | 62.3 | 0 | 274-287 | K.VHGDQPFDYIGFGR.N |  |
| **30** | 1 | 612.3000 | -27.70 | 2 | 11.7 | 62.1 | 1 | 338-348 | R.YINGKDIDGTK.X |  |
| **21** | 1 | 514.3000 | 137.49 | 2 | 11.4 | 26.2 | 0 | 349-357 | K.MSDNNVGYK.N |  |
| **3** | 1 | 522.3000 | 108.64 | 2 | 9.8 | 39.5 | 0 | 349-357 | K.FSDNNVGYK.N |  |
| **9** | 1 | 530.2000 | -76.80 | 2 | 10.2 | 35.8 | 0 | 349-357 | K.YSDNNVGYK.N |  |
| **12** | 1 | 474.8000 | 81.95 | 2 | 10.9 | 44.9 | 0 | 376-384 | K.YVVQSGPAK.D |  |
| **187** | 1 | 523.0000 | 100.70 | 3 | 16.9 | 36.8 | 1 | 376-389 | K.YVVQSGPAKDLSFR.I |  |
| **43** | 2 | 775.8000 | -34.72 | 2 | 12.1 | 59.4 | 0 | 397-410 | R.ANADQGEGDQNEFR.L |  |

Figure I. Peptide mass fingerprint search result of the selected protein spot labelled in section 1.1 (**figure A)** with 599.
