# Supplementary material for: Insights into Mechanisms and Proteomic Characterisation of Pseudomonas aeruginosa Adaptation to a Novel Antimicrobial Substance
Source: PLoS One. 2013 Jul 15;8(7):e66862. doi: 10.1371/journal.pone.0066862 (PMC3711899; doi:10.1371/journal.pone.0066862)
Supplement: File S2 — Time course of adaptation of P. aeruginosa PAO1 in LB medium with increasing concentrations of zinc Schiff-base. (DOC) [file pone.0066862.s002.doc]

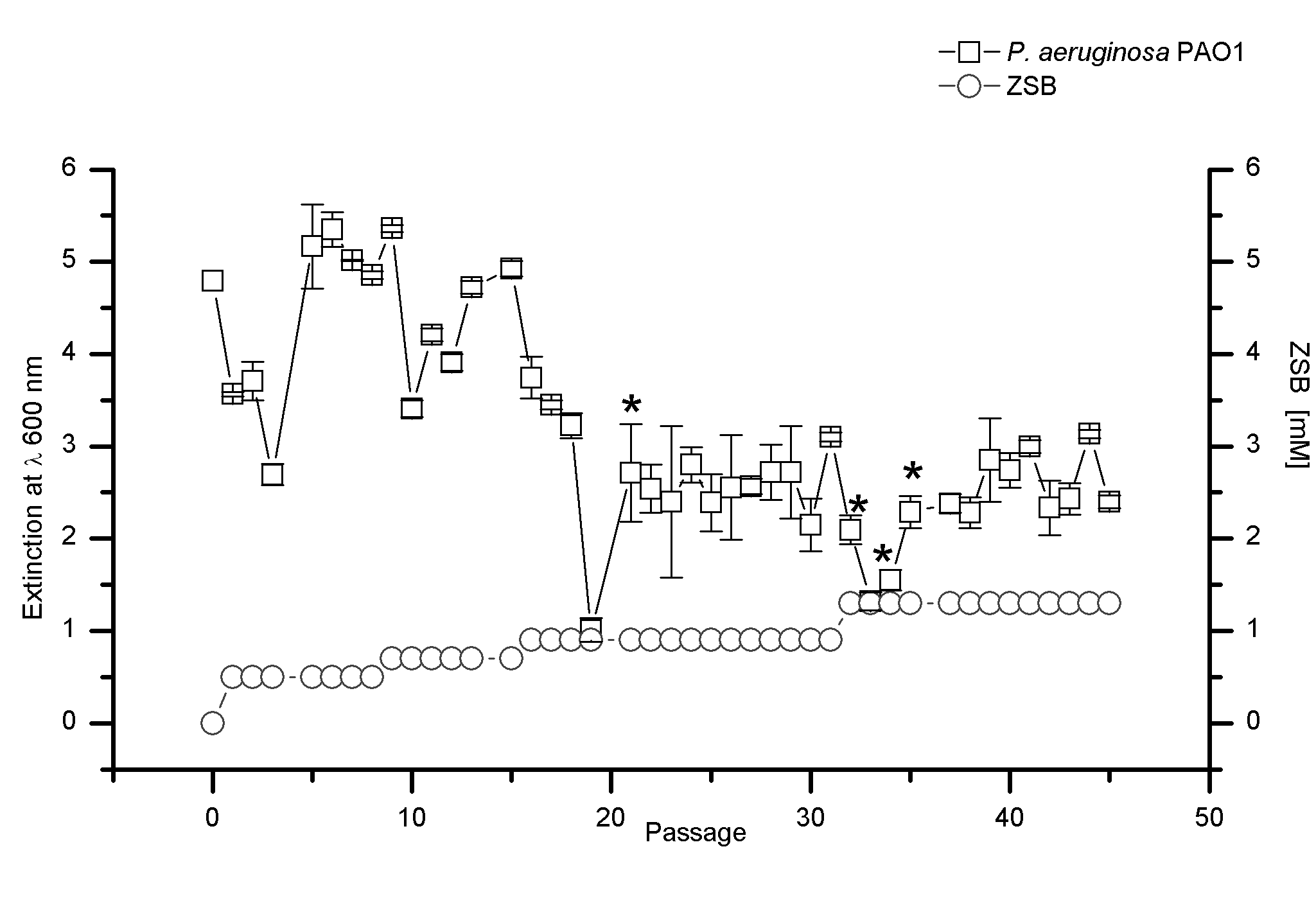


**Figure S2. Time course of adaptation of *P. aeruginosa* PAO1 in LB medium with increasing concentrations of zinc Schiff-base**. ZSB concentration was increased when the cultures showed good recovery. Data points marked with * represent passages incubated for 48 h instead of 20 h. Results from two different cultures are shown. Data points are represented as arithmetic mean values, errors are shown as maximal error from the mean value. Invisible error bars lay within the data points.
