# Supplementary material for: Insights into Mechanisms and Proteomic Characterisation of Pseudomonas aeruginosa Adaptation to a Novel Antimicrobial Substance
Source: PLoS One. 2013 Jul 15;8(7):e66862. doi: 10.1371/journal.pone.0066862 (PMC3711899; doi:10.1371/journal.pone.0066862)
Supplement: File S3 — Single channel image of a two-dimensional difference gel and peptide mass fingerprints. (DOC) [file pone.0066862.s003.doc]

**3.1 Representative two-dimensional difference gel of comparative proteome analysis of ZSB adapted (P_45_) and non-adapted (P_0_) *P. aeruginosa* PAO1 strains.**

(Please note that the difference in appearance in the 2 DE gel between supplementary information figure A in File S3 and figure A in File S1 results from differences in sample preparation procedure)


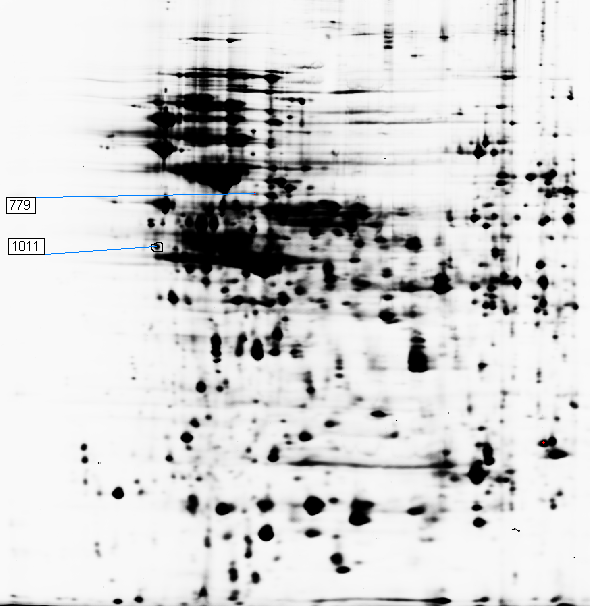


**Figure A. Single channel image of a two-dimensional difference gel with a pH range from pH 4-7, 18 cm. Proteins of significantly differently abundance (ANNOVA p < 0.01, q < 0.01) are marked with a red dot. The selected proteins are annotated with numbers.**

3.2 Detailed Protein Report of significantly different abundant proteins in the of comparative proteome analysis of ZSB adapted and non-adapted *P. aeruginosa* PAO1 strains.

Figures below show the peptide mass fingerprints (PMF) of protein spots highlighted in Figure A. For all selected protein spots only the protein with the highest score is shown.

**Alkyl hydroperoxide reductase subunit F, *Pseudomonas aeruginosa* PAO1**

Accession: gi|15595338 Score: 560.14

Database: NCBInr(NCBInr_20091130.fasta) MW: 55.80 kDa

Database Date: 2009-12-04 pI: 5.12

Modification(s): Oxidation Sequence Coverage: 28.21 %

No. of unique Peptides: 12

**
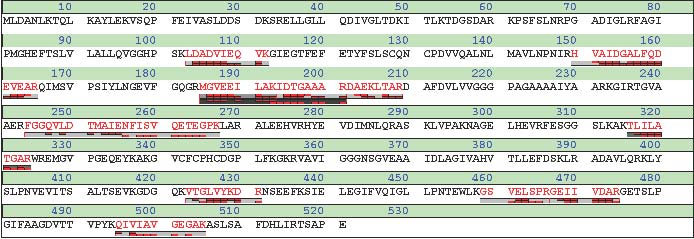
**

| **Cmpd.** | **No. of Cmpds.** | **m/z meas.** | **∆ m/z [ppm]** | **z** | **Rt [min]** | **Score** | **P** | **Range** | **Sequence** | **Modification** |
| --- | --- | --- | --- | --- | --- | --- | --- | --- | --- | --- |
| **154** | 1 | 565.3000 | -15.219 | 2 | 16.03 | 34.79 | 0 | 103-112 | K.LDADVIEQVK.G |  |
| **212** | 1 | 885.9000 | 514.451 | 2 | 17.85 | 80.56 | 0 | 150-165 | R.HVAIDGALFQDEVEAR.Q |  |
| **129** | 1 | 503.3000 | 63.797 | 2 | 15.04 | 54.03 | 0 | 185-193 | R.MGVEEILAK.I | Oxidation: 1 |
| **269** | 1 | 587.7000 | 93.217 | 3 | 19.85 | 34.35 | 1 | 185-201 | R.MGVEEILAKIDTGAAAR.D | Oxidation: 1 |
| **2** | 1 | 387.7000 | -22.851 | 2 | 8.66 | 29.94 | 0 | 194-201 | K.IDTGAAAR.D |  |
| **4** | 1 | 452.3000 | 114.21 | 2 | 8.76 | 35.96 | 1 | 202-209 | R.DAEKLTAR.D |  |
| **379** | 1 | 876.1000 | 5.683 | 3 | 23.75 | 36.60 | 0 | 244-267 | R.FGGQVLDTMAIENFISVQETEGPK.L | Oxidation: 9 |
| **113** | 1 | 458.3000 | 33.31 | 2 | 14.49 | 61.15 | 0 | 316-324 | K.TLILATGAR.W |  |
| **35** | 1 | 525.8000 | -1.424 | 2 | 11.50 | 28.87 | 1 | 423-431 | K.VTGLVYKDR.N |  |
| **29** | 1 | 422.8000 | 166.082 | 2 | 11.26 | 39.61 | 0 | 459-466 | K.GSVELSPR.G |  |
| **68** | 1 | 436.7000 | -104.047 | 2 | 12.94 | 65.72 | 0 | 467-474 | R.GEIIVDAR.G |  |
| **125** | 1 | 542.8000 | -39.941 | 2 | 14.89 | 23.35 | 0 | 495-505 | K.QIVIAVGEGAK.A |  |

**Figure B.** Peptide mass fingerprint search result of the selected protein spot labelled in section 3.1 (**figure A)** with 779.

**Protein 1:** **Chain A, crystal structure of the outer membrane protein Oprd from *Pseudomonas aeruginosa***

Accession: gi|158429225 Score: 778.98

Database: NCBInr(NCBInr_20091130.fasta) MW: 46.97 kDa

Database Date: 2009-12-04 pI: 5.28

Modification(s): Oxidation Sequence Coverage: 30.37 %

No. of unique Peptides: 14

**
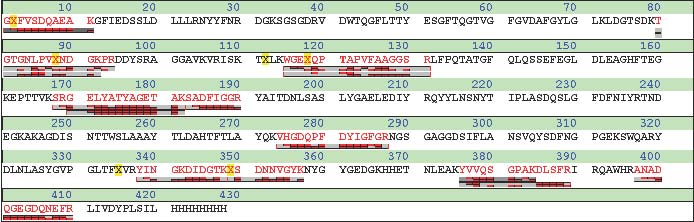
**

| **Cmpd.** | **No. of Cmpds.** | **m/z.** | **∆ m/z [ppm]** | **z** | **Rt [min]** | **Score** | **P** | | **Range** | **Sequence** | **Modification** |
| --- | --- | --- | --- | --- | --- | --- | --- | --- | --- | --- | --- |
| **97** | 1 | 590.7000 | -131.466 | 2 | 11.85 | 82.73 | 0 | 1-11 | | -.GEFVSDQAEAK.G |  |
| **70** | 1 | 660.3000 | -50.189 | 2 | 10.93 | 26.49 | 0 | 80-92 | | K.TGTGNLPVFNDGK.P |  |
| **58** | 1 | 786.8000 | -118.912 | 2 | 10.50 | 37.42 | 1 | 80-94 | | K.TGTGNLPVMNDGKPR.D | Oxidation: 9 |
| **243** | 1 | 889.4000 | -22.434 | 2 | 16.72 | 55.58 | 0 | 115-131 | | K.WGEMQPTAPVFAAGGSR.L | Oxidation: 4 |
| **240** | 1 | 593.3000 | 11.126 | 3 | 16.77 | 28.35 | 0 | 115-131 | | K.WGEFQPTAPVFAAGGSR.L |  |
| **134** | 1 | 808.9000 | 0.771 | 2 | 13.37 | 78.49 | 1 | 168-182 | | K.SRGELYATYAGETAK.S |  |
| **168** | 1 | 687.3000 | -47.731 | 2 | 14.23 | 102.15 | 0 | 170-182 | | R.GELYATYAGETAK.S |  |
| **118** | 1 | 411.7000 | -21.519 | 2 | 12.84 | 47.89 | 0 | 183-190 | | K.SADFIGGR.Y |  |
| **304** | 1 | 804.4000 | 20.268 | 2 | 18.61 | 46.58 | 0 | 274-287 | | K.VHGDQPFDYIGFGR.N |  |
| **53** | 1 | 612.3000 | -27.698 | 2 | 10.44 | 39.81 | 1 | 338-348 | | R.YINGKDIDGTK.X |  |
| **15** | 1 | 522.2000 | -82.842 | 2 | 8.76 | 38.29 | 0 | 349-357 | | K.FSDNNVGYK.N |  |
| **30** | 1 | 474.8000 | 81.953 | 2 | 9.62 | 44.14 | 0 | 376-384 | | K.YVVQSGPAK.D |  |
| **191** | 1 | 783.9000 | -22.161 | 2 | 15.06 | 42.64 | 1 | 376-389 | | K.YVVQSGPAKDLSFR.I |  |
| **71** | 1 | 775.8000 | -34.724 | 2 | 10.97 | 97.05 | 0 | 397-410 | | R.ANADQGEGDQNEFR.L |  |

**Protein 2:** **Basic amino acid, basic peptide and imipenem outer membrane porin OprD precursor, *Pseudomonas aeruginosa* PAO1**

Accession: gi|15596155 Score: 734.09

Database: NCBInr(NCBInr_20091130.fasta) MW:48.33 kDa

Database Date: 2009-12-04 pI: 4.82

Modification(s): Oxidation Sequence Coverage: 29.12 %

No. of unique Peptides: 1

**
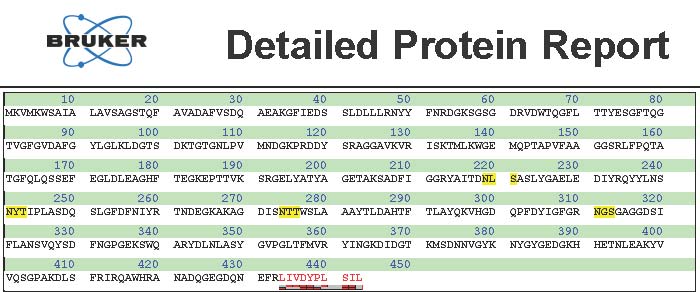
**

| **Cmpd.** | **No. of Cmpds.** | **m/z meas.** | **∆ m/z [ppm]** | **z** | **Rt [min]** | **Score** | **P** | **Range** | **Sequence** | **Modification** |
| --- | --- | --- | --- | --- | --- | --- | --- | --- | --- | --- |
| 527 | 1 | 573.3000 | -77.541 | 2 | 24.97 | 58.45 | 0 | 434-443 | R.LIVDYPLSIL |  |

**Figure C.** Peptide mass fingerprint search result of the selected protein spot labelled in section 3.1 (**figure A)** with 1011. Please note that for this protein spot the first two probable proteins are shown.
