# Supplementary material for: Insights into Mechanisms and Proteomic Characterisation of Pseudomonas aeruginosa Adaptation to a Novel Antimicrobial Substance
Source: PLoS One. 2013 Jul 15;8(7):e66862. doi: 10.1371/journal.pone.0066862 (PMC3711899; doi:10.1371/journal.pone.0066862)
Supplement: File S4 — Measurement of intracellular hydrogenperoxide formation using 2,7-dichlorodihydro-fluorescein diacetate. (DOC) [file pone.0066862.s004.doc]

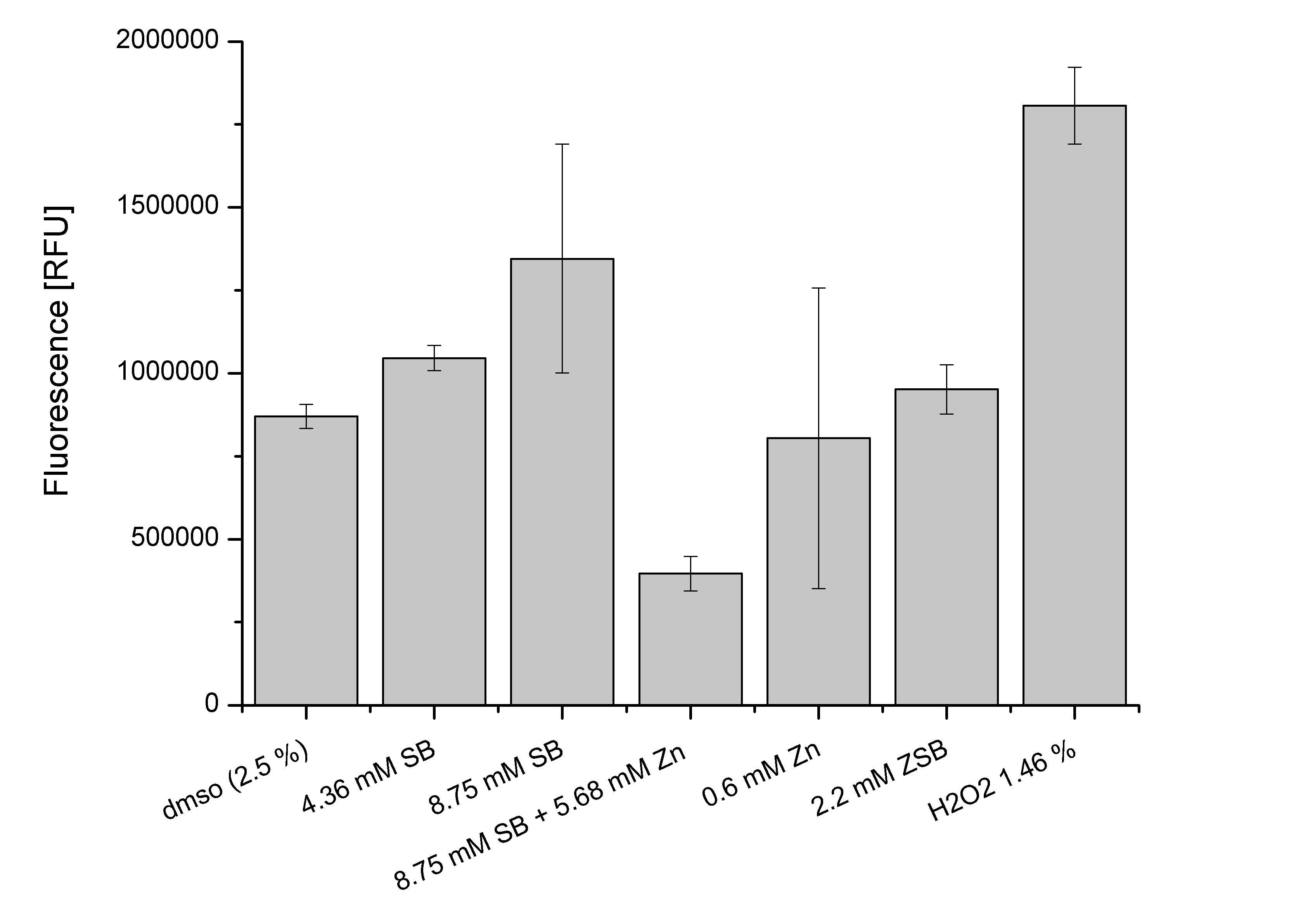


**Figure S4. Measurement of intracellular hydrogenperoxide formation using 2,7‑dichlorodihydro-fluorescein diacetate.** *P. aeruginosa* PAO1 P_0_ was grown to an optical density of 2.8, the culture was then incubated with 2,7-dichlorodihydro-fluorescein after which one of the following was added: Schiff’s Base (SB) in different concentrations (4.36 mM SB, 8.75 mM SB), ZSB (2.2 mM ZSB), zinc as Zn acetate alone (0.6 mM Zn); or Zn together with SB (8.75 mM SB + 5.68 mM Zn). A solution of 2.5 % DMSO was used as a negative control and 1.46 % hydrogen peroxide (H_2_O_2_) as a positive control. Please note that one mole ZSB is equivalent to 4 mole of SB. As shown, the amount of intracellular hydrogen peroxide increases with increasing SB concentration. If SB is added together with Zn, the signal for hydrogen peroxide decreases below the signal of the DMSO control. Cultures grown with ZSB show a fluorescence signal intensity lying between the signal intensity measured with Zn + SB and the SB, respectively. After plating out, a significantly lower number of colony forming units could be found only for the 8.75 mM SB treated cultures (n=8).
